# Whole Transcriptome Sequencing Analysis of the Synergistic Antimicrobial Effect of Metal Oxide Nanoparticles and Ajoene on *Campylobacter jejuni*

**DOI:** 10.3389/fmicb.2018.02074

**Published:** 2018-08-31

**Authors:** Rui Xue, Jinsong Feng, Lina Ma, Chunrong Liu, Ming Xian, Michael E. Konkel, Shuo Wang, Xiaonan Lu

**Affiliations:** ^1^Tianjin Key Laboratory of Food Science and Health, School of Medicine, Nankai University, Tianjin, China; ^2^Food, Nutrition, and Health Program, Faculty of Land and Food Systems, The University of British Columbia, Vancouver, BC, Canada; ^3^Key Laboratory of Food Nutrition and Safety, Ministry of Education of China, Tianjin University of Science and Technology, Tianjin, China; ^4^Department of Chemistry, Washington State University, Pullman, WA, United States; ^5^School of Molecular Biosciences, College of Veterinary Medicine, Washington State University, Pullman, WA, United States

**Keywords:** *Campylobacter*, RNA-seq, organosulfur compound, Al_2_O_3_ nanoparticle, TiO_2_ nanoparticle

## Abstract

Two metal oxide (i.e., Al_2_O_3_ and TiO_2_) nanoparticles and ajoene, a garlic-derived organosulfur compound, were identified to be effective antimicrobials against *Campylobacter jejuni*, a leading cause of human gastrointestinal diseases worldwide. A significant synergistic antimicrobial effect was observed using ajoene and Al_2_O_3_/TiO_2_ nanoparticles in a combined manner to cause at least 8 log_10_ CFU/mL reduction of *C. jejuni* cells. Whole transcriptome sequencing (RNA-seq) and confocal micro-Raman spectroscopic analyses revealed the antimicrobial mechanism and identified the roles of ajoene and metal oxide nanoparticles in the synergistic treatment. Ajoene and metal oxide nanoparticles mediated a two-phase antimicrobial mechanism. Ajoene served as the inducing factor at the first phase that caused injury of cell membranes and increased the susceptibility of *C. jejuni* to stress. Metal oxide nanoparticles served as the active factor at the second phase that targeted sensitive cells and physically disrupted cell structure. This synergistic antimicrobial treatment demonstrates a potential to reduce the prevalence of *C. jejuni* and other pathogens on food contact surfaces and in the food chain.

## Introduction

Metal oxide nanoparticles have been widely used in human daily life. For example, aluminum oxide (Al_2_O_3_) nanoparticles have wide-range applications in industrial as well as personal care products. Titanium dioxide (TiO_2_) is a common additive in many personal care commodities (e.g., toothpaste), pharmaceuticals, and food products, such as chewing gums and candies (Weir et al., 2012). Several recent studies have demonstrated that metal oxide nanoparticles exhibited antimicrobial effects against different pathogenic bacterial cells and biofilms, such as *Salmonella*, *Escherichia coli* O157:H7, *Listeria monocytogenes*, and *Campylobacter*, due to their unique electrical, chemical, and physical properties (Chen et al., 2014; Ertem et al., 2017; Fathima et al., 2017). However, treatment with metal oxide nanoparticles may fail to completely inactivate pathogens. For example, *Cupriavidus metallidurans* was found to over-synthesize membrane protective and restoration elements to neutralize the damage caused by the treatment of TiO_2_ nanoparticles (Simon-Deckers et al., 2009). In addition, the use of metal oxide nanoparticle was found to potentially induce the emergence of resistance. For example, Al_2_O_3_ nanoparticles were found to increase the conjugative transfer of antibiotic-resistant genes RP4 plasmid from *E. coli* to *Salmonella* by up to 200-fold (Qiu et al., 2012). Alternative strategies that can complement to metal oxide nanoparticle will expand the antimicrobial application.

Ajoene is an organosulfur compound derived from oil-macerated or ether-extracted garlic oil. Previous studies validated its significant antimicrobial effect against *Cronobacter sakazakii* (Feng et al., 2014), *Pseudomonas aeruginosa* (Fong et al., 2016), *Helicobacter pylori* (Ohta et al., 1999), *Bacillus cereus* and *Bacillus subtilis* (Naganawa et al., 1996), and *Staphylococcus aureus* (Yoshida et al., 1998). Ajoene may also be useful as an antimicrobial drug to combat bacterial illnesses. For example, ajoene was used as an antimicrobial complement with tobramycin to clear *P. aeruginosa* infection in mice (Jakobsen et al., 2012). Compared to other garlic-derived thiosulfinates, ajoene demonstrates a high biological and chemical stability, showing the potential to be applied in food products to maintain a persistent antimicrobial effect (Ankri and Mirelman, 1999).

Transcriptome analysis can provide valuable information about the bacterial responses to stress. Recent studies have applied transcriptome analysis to investigate the response of various pathogens (e.g., *E. coli* O157:H7, *L. monocytogenes*, and *Salmonella*) exposed to the oxidative stress (Wang et al., 2009), inorganic and organic acids (King et al., 2010), lysates of lettuce leaves (Kyle et al., 2010), chlorine dioxide (Pleitner et al., 2014), hyperosmotic and low temperature (Durack et al., 2013), dehydration (Gruzdev et al., 2012), starvation in peanut oil (Deng et al., 2012), and chlorine (Wang et al., 2010).

In the current study, we tested the individual and combinatorial antimicrobial effect of ajoene and metal oxide nanoparticles (Al_2_O_3_ and TiO_2_ nanoparticles) against *C. jejuni*, a leading bacterial cause of human gastroenteritis. The mechanism of stress and sub-lethal injury of *C. jejuni* by the aforementioned individual and combinatorial antimicrobial treatments were studied using high-throughput whole transcriptome sequencing (RNA-seq) and confocal micro-Raman spectroscopic analyses. The knowledge from this study can aid the development of innovative antimicrobial treatments to reduce campylobacteriosis and potentially other foodborne illnesses.

## Materials and Methods

### Chemicals and Reagents

Al_2_O_3_ nanoparticles with the size of 30–60 nm and TiO_2_ nanoparticles with the size of 21 nm were purchased from Sigma-Aldrich (St Louis, MO, United States). Ajoene was synthesized according to the protocols described in our previous study (Feng et al., 2014). Diallyl disulfide was used as a precursor to synthesize ajoene. Briefly, diallyl disulfide solution (Sigma-Aldrich; 80% in chloroform; 2.1 mL, 11.3 mM) was reacted with peracetic acid (35%; 2.58 g, 11.87 mM) at 0°C for 30 min to receive allicin. Allicin (1 g, 6.2 mM) was then reacted with 40% aqueous acetone (10 mL) at 65°C for 4 h. The reaction mixture was diluted with aqueous methanol (50%, 40 mL) and washed with hexane (five times volume; 20 mL). Ajoene in the methanolic aqueous layer was then extracted with methylene chloride (20 times volume; 2 mL) and purified by flash chromatography.

### Bacterial Strains and Culture Methods

Four strains of *C. jejuni* (F38011, ATCC 33560, y110539, and z110526) were used in this study. The description about these *C. jejuni* strains is listed in **Supplementary Table S1**. All the strains were stored at -80°C in Mueller–Hinton (MH) broth containing 75% citrated bovine blood and 12% glycerol. *C. jejuni* strains were routinely cultivated either on MH agar plates supplemented with 5% citrated bovine blood or in 5 mL of MH broth at 37°C overnight under a microaerobic condition (85% N_2_, 10% CO_2_, 5% O_2_). One milliliter of each *C. jejuni* culture (∼ca. 9 log10 CFU/mL) was centrifuged at 8,000 × *g* for 10 min at 4°C. The supernatant was discarded and *C. jejuni* pellets were washed three times and resuspended in the sterile MH broth. An equal volume of each *C. jejuni* culture was combined as a cocktail with an initial concentration of ∼8 log10 CFU/mL for the subsequent antimicrobial tests.

### Antibacterial Effects of Metal Oxide Nanoparticles and Ajoene Against *C. jejuni*

The Al_2_O_3_ nanoparticle suspension (20% w/v in H_2_O) and TiO_2_ nanoparticles (in a powder form) were diluted separately with sterile deionized water to the initial concentration of 1 M and stored at 22°C. Ajoene was dissolved in DMSO to the initial concentration of 0.1 M and stored at 4°C. The antimicrobial effect of ajoene was tested by challenging *C. jejuni* culture with ajoene at a series of concentrations from 0.06, 0.125, 0.25, 0.5, to 1 mM. The antimicrobial effect of metal oxide nanoparticle was tested by challenging *C. jejuni* cocktail culture with metal oxide nanoparticle at a series of concentrations from 0.5, 1, 2, 4, 8, and 16 mM. In addition, metal oxide nanoparticle suspensions were filtered through an aluminum oxide membrane filter (20 nm pore size, 25 mm optical density, Anodisc; Whatman Inc., Clifton, NJ, United States) to harvest a nanoparticle-free solution containing surfactants. *C. jejuni* culture with the addition of nanoparticle-free solution and *C. jejuni* culture with the addition of DMSO (1 mM) were used as the control groups. The treatment was conducted with constant shaking (175 rpm) in a microaerobic condition at both 22 and 37°C for up to 24 h. At 0, 2, 4, 7, 10, and 24 h, viable *C. jejuni* cells were enumerated on MH blood agar plates.

### Synergistic Antibacterial Effect of Metal Oxide Nanoparticle and Ajoene on *C. jejuni*

Ajoene at the concentration of 0.06 mM was combined with different concentrations (i.e., 0, 0.5, 1, 2, 4, 8, and 16 mM) of either Al_2_O_3_ nanoparticles or TiO_2_ nanoparticles for the test of synergistic antimicrobial effect against *C. jejuni* cocktail. The treatment was conducted under constant shaking (175 rpm) in a microaerobic condition at both 22 and 37°C for up to 24 h. At 0, 2, 4, 7, 10, and 24 h, viable *C. jejuni* cells were enumerated on MH blood agar plates.

### RNA-seq and Real-Time Polymerase Chain Reaction (qPCR)

*Campylobacter jejuni* F38011 (OD_540_ ∼0.3) was treated with 1 mM ajoene, 16 mM Al_2_O_3_ nanoparticles, 16 mM TiO_2_ nanoparticles, a combination of 0.06 mM ajoene and 4 mM Al_2_O_3_ nanoparticles, and a combination of 0.06 mM ajoene and 4 mM TiO_2_ nanoparticles, respectively, for 1 h at 37°C in microaerobic conditions with constant shaking (175 rpm). The untreated and treated samples were collected by centrifugation at 8,000 × *g* for 5 min at 4°C. The total RNA was extracted using a RiboPure^TM^ RNA purification kit (Life Technologies, Grand Island, NY, United States). The rRNA was removed using a MICROBExpress^TM^ bacterial mRNA enrichment kit (Life Technologies, Grand Island, NY, United States). The purified mRNA was sequenced using an Ion Torrent sequencing system (Life Technologies) and the data were compiled and analyzed using CLC genomics workbench software (CLCBio, Cambridge, MA, United States). The analyzed transcriptomes were sorted by false discovery rate (FDR)-adjusted *P*-values of less than 0.05 and a relative expression change of greater than twofold. The differentially expressed genes were submitted to DAVID for cluster analysis (Huang et al., 2008).

The RNA-seq result was validated using quantitative PCR (qPCR). An aliquot of the purified mRNA was used to generate cDNA using SuperScript^TM^ II Reverse Transcriptase (Invitrogen). The qPCR was performed using cDNA derived from the samples with Power SYBR green PCR Master Mix (Applied Biosystems, Warrington, United Kingdom) on an ABI Prism 7000 Fast instrument (Life Technologies). The primers used for qPCR validation are listed in **Supplementary Table S2**. Both *gyrA* and *rpoA* (housekeeping genes) were used as the internal controls. The result of qPCR was accepted with an amplification efficiency ranged from 90 to 110%. The fold change of different genes was determined using the comparative Ct method as previously described (Schmittgen and Livak, 2008).

### Confocal Micro-Raman Spectroscopy

A confocal micro-Raman spectroscopic system (Renishaw, Gloucestershire, United Kingdom) with a 785-nm diode near-infrared laser was used to investigate the change of biochemical compositions of *C. jejuni* F38011 cells after the antimicrobial treatment. *C. jejuni* strain F38011 (OD_540_ ∼0.3) was treated with 1 mM ajoene, 16 mM Al_2_O_3_ nanoparticles, 16 mM TiO_2_ nanoparticles, a combination of 0.06 mM ajoene and 4 mM Al_2_O_3_ nanoparticles, and a combination of 0.06 mM ajoene and 4 mM TiO_2_ nanoparticles, respectively, for 1 h at 37°C in microaerobic conditions. The untreated and treated samples were washed three times with sterilized water and collected by centrifugation at 8,000 × *g* for 5 min at 4°C. The bacterial pellet was individually deposited on a gold-coated microarray chip (BioGold^TM^, Thermo Scientific^TM^, Waltham, United States) and air-dried for 30 min. The chip with bacterial samples was then mounted on the microscope stage and Raman laser was introduced onto the samples through a 50 × objective (numerical aperture [NA] = 0.75, working distance [WD] = 0.37 mm, Leica Biosystems, Wetzlar, Germany). The working power of Raman laser was ∼0.2 mW. Raman scattering signal was recorded using a 578-pixel by 384-pixel charge-coupled device (CCD) array detector. The integration time was set as 10 s over a simultaneous Raman shift range from 400 to 1,800 cm^-1^. Raman spectrometer was controlled via WiRE software for spectral acquisition and processing (Renishaw, Wotton-under-Edge, United Kingdom). Raman laser exposure did not cause damage to the bacterial samples as no spectral variation occurred during Raman spectral collection (data not shown). The polynomial background fit and baseline subtraction were applied to the collected raw Raman spectra to remove the fluorescence background, followed by spectral binning (2 cm^-1^) and smoothing (nine-point Savitzky–Golay algorithm; Feng et al., 2014). To identify the minor difference among Raman spectra of bacterial cells with different antimicrobial treatments, a second derivative transformation algorithm of the processed Raman spectra was applied (Lu et al., 2011).

### Scanning Electron Spectroscopy

Scanning electron microscopy (SEM) was applied to investigate the interaction between metal oxide nanoparticles and *C. jejuni* cells. *C. jejuni* strain F38011 (OD_540_ ∼0.3) was treated with 1 mM ajoene, 16 mM Al_2_O_3_ nanoparticles, 16 mM TiO_2_ nanoparticles, a combination of 0.06 mM ajoene and 4 mM Al_2_O_3_ nanoparticles, and a combination of 0.06 mM ajoene and 4 mM TiO_2_ nanoparticles, respectively, for 1 h at 37°C in microaerobic conditions. The untreated and treated bacterial samples were washed three times with sterilized water and collected by centrifugation at 8,000 × *g* for 10 min at 4°C. Bacteria pellet was individually fixed with 2.5% (w/v) glutaraldehyde at 4°C for overnight. The samples were then rinsed twice with 0.1 M phosphate buffer, dehydrated in a series of concentrations of ethanol (25, 50, 70, 80, 90, 10 min for each concentration) and post-fixed using 1% (w/v) osmium tetroxide for 1 h. The reaction mixture was freeze-dried in a lyophilizer (Christ, Osterode, Germany). The freeze-dried samples were coated with a layer of gold and then examined by a scanning electron microscope with an accelerating voltage of 5 kV (Leo 1530 Gemini, Zeiss, Jena, Germany).

### Statistical Analysis

All the experiments were performed at least three times. The results are expressed as the mean value of three independent biological replicates ± the standard deviations. The differences between groups are shown to be significant (*P* < 0.05) by one-way analysis of variance (ANOVA) using the Matlab software.

## Results

### Antibacterial Effect of Ajoene Against *C. jejuni*

**FIGURE 1:**
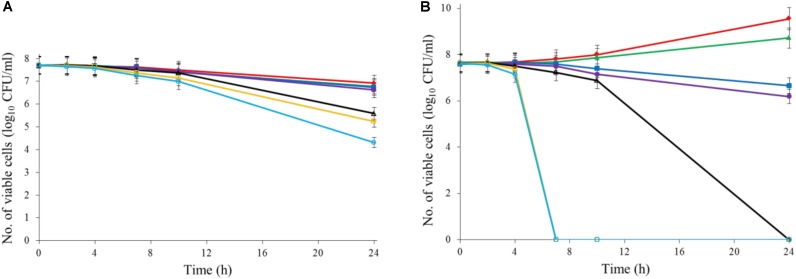
Ajoene treatment on *C. jejuni* cocktail (the mixture of *C. jejuni* strain F38011, ATCC 33560, y110539, and z110526) resulted in a bacteriostatic effect at 22°C **(A)** and a bactericidal effect at 37°C **(B)**. Different symbols indicate different concentrations of ajoene (red line: 0 mM; green line: DMSO; blue line: 0.06 mM; purple line: 0.125 mM; black line: 0.25 mM; orange line: 0.5 mM; light blue line: 1 mM) (*n* = 3).

Ajoene demonstrated a concentration-dependent antimicrobial effect against *C. jejuni* at both 22 and 37°C (**Figure 1**). The ajoene treatment at low concentration could only generate a bacteriostatic effect. When the concentration was higher than 0.25 mM, ajoene treatment could significantly reduce cell viability. In addition, we found that the antimicrobial effect of ajoene was influenced by temperature. The antimicrobial effect of ajoene at 37°C was much higher than that at 22°C. For example, ajoene treatment at concentration of 1 mM could generate a ∼7 log_10_ CFU/mL reduction of viable *C. jejuni* cells within 7 h at 37°C. In contrast, the treatment at the same concentration could only generate ∼3 log_10_ CFU/mL reduction of viable *C. jejuni* cells at 22°C for 24 h. We speculated that ajoene treatment would be more likely to inactivate bacterial cells with high metabolic activities.

### Antibacterial Effect of Metal Oxide Nanoparticles Against *C. jejuni*

**FIGURE 2:**
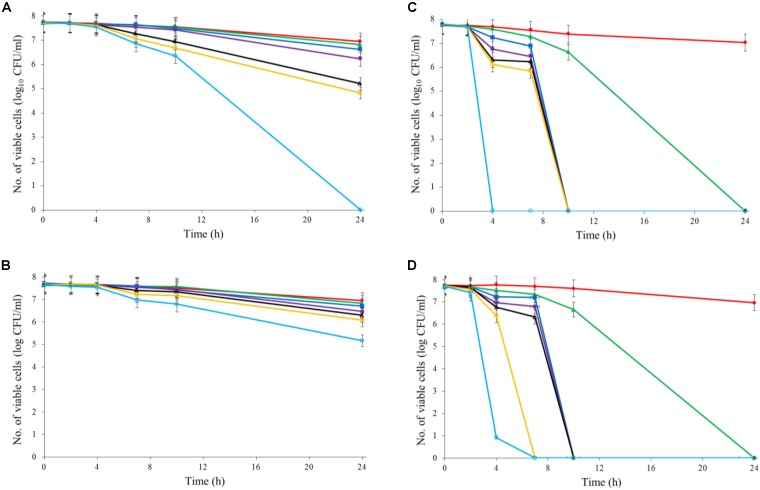
Synergistic antimicrobial effect of ajoene and metal oxide nanoparticles against *C. jejuni* cocktail (the mixture of *C. jejuni* strain F38011, ATCC 33560, y110539, and z110526) at 22°C. **(A)** Antimicrobial effect of TiO_2_ nanoparticles. **(B)** Antimicrobial effect of Al_2_O_3_ nanoparticles. **(C)** Synergistic antimicrobial effect of 0.06 mM ajoene and TiO_2_ nanoparticles. **(D)** Synergistic antimicrobial effect of 0.06 mM ajoene and Al_2_O_3_ nanoparticles. Different symbols indicate different concentrations of metal oxide nanoparticles (red line: 0 mM; green line: 0.5 mM; blue line: 1 mM; purple line: 2 mM; black line: 4 mM; orange line: 8 mM; light line: 16 mM) (*n* = 3).

**FIGURE 3:**
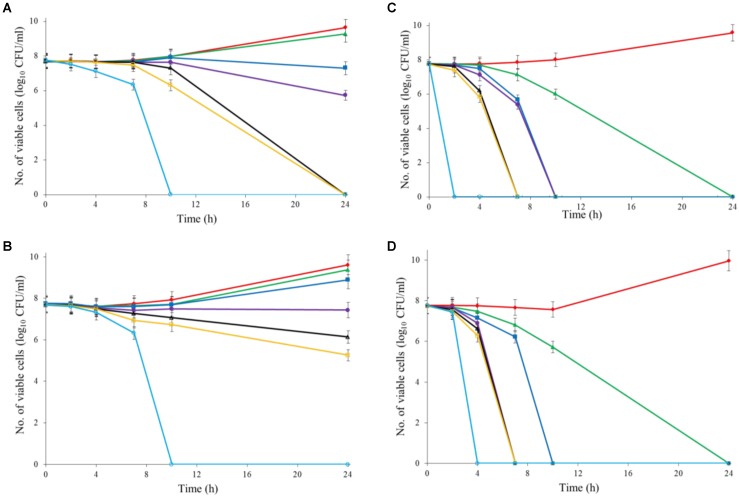
Synergistic antimicrobial effect of ajoene and metal oxide nanoparticles against *C. jejuni* cocktail (the mixture of *C. jejuni* strain F38011, ATCC 33560, y110539, and z110526) at 37°C. **(A)** Antimicrobial effect of TiO_2_ nanoparticles. **(B)** Antimicrobial effect of Al_2_O_3_ nanoparticles. **(C)** Synergistic antimicrobial effect of 0.06 mM ajoene and TiO_2_ nanoparticles. **(D)** Synergistic antimicrobial effect of 0.06 mM ajoene and Al_2_O_3_ nanoparticles. Different symbols indicate different concentrations of metal oxide nanoparticles (red line: 0 mM; green line: 0.5 mM; blue line: 1 mM; purple line: 2 mM; black line: 4 mM; orange line: 8 mM; light blue line: 16 mM) (*n* = 3).

Both Al_2_O_3_ and TiO_2_ nanoparticles could inactivate *C. jejuni* and the antimicrobial effect was concentration dependent. TiO_2_ nanoparticles exhibited a better antimicrobial effect against *C. jejuni* than Al_2_O_3_ nanoparticles did (**Figures 2A,B, 3A,B**). The TiO_2_ nanoparticle treatment at the concentration of 16 mM could completely inactivate *C. jejuni* cells (∼8 log10 CFU/mL reduction) at 22°C within 24 h. In contrast, Al_2_O_3_ nanoparticle treatment at the same conditions could only generate ∼2 log_10_ CFU/mL reduction of *C. jejuni* cells. Similar to ajoene, the antimicrobial effect of both Al_2_O_3_ and TiO_2_ nanoparticles was influenced by temperature (**Figures 2A,B, 3A,B**). The high temperature could enhance the antimicrobial efficacy. For example, TiO_2_ nanoparticle treatment at 16 mM could generate a ∼8 log_10_ CFU/mL reduction of *C. jejuni* cells within 10 h at 37°C (**Figure 3A**), whereas it would require at least 24 h to achieve the same antimicrobial effect at 22°C (**Figure 2A**).

### Synergistic Antimicrobial Effect of Metal Oxide Nanoparticle and Ajoene Against *C. jejuni*

The metal oxide nanoparticles with ajoene could generate a strong antimicrobial effect against *C. jejuni* (**Figures 2C, 3D**). Synergy was achieved when the concentration of Al_2_O_3_ and TiO_2_ nanoparticles was 0.05 mM or higher, which could lead to the complete inactivation of *C. jejuni* within 24 h. In addition, the antimicrobial efficacy of combinatorial treatment was enhanced by the increase in the concentration of metal oxide nanoparticles. For example, combinatorial treatment with 1 mM of Al_2_O_3_ or TiO_2_ nanoparticles could achieve a bactericidal effect within 10 h. In comparison, the same antimicrobial effect can be achieved within 4 h when the concentration of Al_2_O_3_ or TiO_2_ nanoparticles was increased to 16 mM.

Compared to the individual treatment of ajoene or metal oxide nanoparticles, the antimicrobial effect of combinatorial treatment was not influenced by temperature. We speculated that combinatorial treatment already achieved the maximum antimicrobial effect. Hence, a higher temperature that enhanced the antimicrobial effect of individual treatment did not influence the antimicrobial effect of combinatorial treatment.

### Transcriptome Analysis of the Response of *C. jejuni* to Antimicrobial Treatment

The synergistic antimicrobial mechanism of ajoene and metal oxide nanoparticles against *C. jejuni* was investigated using RNA-seq analysis. *C. jejuni* strain F38011 was used as it is a representative *Campylobacter* clinical isolate. RNAs were extracted from three biological samples of *C. jejuni* after 1-h treatments at 37°C. cDNA libraries were then constructed, and RNA-seq was performed using an Ion Torrent sequencing system. To ensure reproducible results, each RNA-seq experiment was repeated twice, resulting in a total of 12 samples (treatment groups: untreated group, 1 mM ajoene treated group, 16 mM Al_2_O_3_ nanoparticles treated group, 16 mM TiO_2_ nanoparticles treated group, a combination of 0.06 mM ajoene and 4 mM Al_2_O_3_ nanoparticles treated group, and a combination of 0.06 mM ajoene and 4 mM TiO_2_ nanoparticles treated group). Each library yielded an average of 3.9 million total reads (2.8 and 5.1 million reads for each library). The reads were mapped to the reference genome, resulting in an average of 135,000 sequence reads per sample per replicate, giving a coding region sequence (CDS) coverage of 9.5-fold. Additional analysis revealed that every gene was represented in the RNA-seq data (*n* = 1,798 genes). The sequence coverage at the chromosomal and gene levels provided confidence that the data generated were of good quality. The differentially expressed genes derived from different treatment groups are summarized in **Supplementary Table S4** and the accuracy of RNA-seq results was confirmed by qPCR. The expression profiles of selected genes (CJH_02830, CJH_07030, CJH_02225, CJH_03855, CJH_05030, CJH_08660, and CJH_02460) were determined by qPCR and were consistent with the expression levels determined by RNA-seq (**Supplementary Figure S1**).

According to RNA-seq analysis, treatments induced different transcriptional responses. The individual ajoene treatment could induce a range of transcriptional responses in *C. jejuni*, including 34 upregulated genes and 18 downregulated genes. In contrast, the treatments with metal oxide nanoparticles barely induced any detectable transcriptional response in *C. jejuni*. The differentially expressed genes in response to different antimicrobial treatments were categorized based on the functional terms using DAVID analysis (Huang et al., 2009), and the results are listed in **Tables 1, 2**. The upregulated genes in response to individual ajoene treatment were categorized into two functional groups, referring to the term of transcription-translation and the term of ATP utilization (**Supplementary Figures S2A**, S4B). In addition, a set of efflux pump genes, including *cmeA*, *cmeB*, and *cmeC*, two heat shock response genes *dnaK* and *groEL*, and an oxidative stress response gene *katA* were also upregulated (**Supplementary Table S4**). Downregulated genes in response to individual ajoene treatment were clustered in one group that referred to the term of integral cell membrane (**Supplementary Figure S2C**). In addition, a DNA repair-associated gene *recN* and a chemotaxis-associated gene *cheY* were also significantly downregulated (**Supplementary Table S4**). We concluded that individual ajoene treatment induced mild stress that damaged the cell membrane and activated the stress response of *C. jejuni*.

**Table 1:** Differentially expressed genes in *C. jejuni* F38011 cells induced by ajoene treatment.

Regulation category and annotation cluster	Term and function	Fold enrichment	Benjamini FDR
**Upregulation**			
1 (Enrichment score, 11.56)	GO:0019843; rRNA binding	18	0.00
	GO:0003723; RNA binding	16	0.00
	GO:0019843; rRNA binding	14	0.00
	GO:0005840; ribosome	14	0.00
	GO:0030529; intracellular ribonucleoprotein complex	11	0.00
	GO:0005840; ribosome	13	0.00
	GO:0006412; translation	8.7	0.00
	GO:0003735; structural constituent of ribosome	6.9	0.00
	GO:0005840; ribosome	8.1	0.00
	GO:0015934; large ribosomal subunit	22	0.02
2 (Enrichment score, 0.12)	GO:0005524; ATP binding	1.2	1.00
	GO:0000166; nucleotide binding	1	1.00
	GO:0005524; ATP binding	0.8	1.00
**Downregulation**			
1 (Enrichment score, 0.27)	GO:0016021; integral component of membrane	1.5	0.93
	GO:0016021; transmembrane helix	1.3	1.00
	GO:0016021; transmembrane	1.3	1.00
	GO:0005886; plasma membrane	1.2	1.00

*The genes categorized in upregulation group 1 are marked as green in **Supplementary Table S4**; the genes categorized in upregulation group 2 are marked as blue in **Supplementary Table S4**; the genes categorized in downregulation group 1 are marked as red in **Supplementary Table S4**.*

**Table 2:** Differentially expressed genes in *C. jejuni* F38011 cells induced by the treatment of 16 mM TiO_2_ nanoparticles.

Regulation category and annotation cluster	Term and function	Fold enrichment	Benjamini FDR
**Upregulation**			
1 (Enrichment score, 0.89)	GO:0016021; transmembrane helix	3.1	0.70
	GO:0016021; transmembrane	3.1	0.46
	GO:0005886; plasma membrane	2.8	0.42
	GO:0016021; integral component of membrane	1.8	0.57

*The genes categorized in upregulation group 1 are marked as yellow in **Supplementary Table S4**.*

The treatment of Al_2_O_3_ nanoparticles with or without ajoene barely induced any transcriptional response in *C. jejuni*. The individual treatment with 16 mM Al_2_O_3_ nanoparticles induced two differentially expressed genes, namely, CJH_08660 (encoding an integral membrane protein, upregulated by 3.7-fold) and CJH_05030 (encoding a hypothetical periplasmic protein, downregulated by 7.4-fold). The combinatorial treatment of 4 mM Al_2_O_3_ nanoparticles and 0.06 mM ajoene also induced two differentially expressed genes, namely, CJH_03855 (encoding 3-polyprenyl-4-hydroxybenzoate decarboxylase, downregulated by 3.7-fold) and CJH_00775 (encoding hydroxybenzoate decarboxylase, upregulated by 7.8-fold), but there was no overlap with the treatment of Al_2_O_3_ nanoparticles alone.

The individual treatment with 16 mM TiO_2_ nanoparticles induced upregulation of six genes and downregulation of one gene. The upregulated genes were categorized in a functional group that referred to the term of integral cell membrane (**Supplementary Figure S2D**). The combinatorial treatment of 4 mM TiO_2_ nanoparticles and 0.06 mM ajoene only induced one upregulated gene CJH_08660 (encoding an integral membrane protein, upregulated by 3.5-fold).

Although the transcriptional responses of *C. jejuni* to ajoene and metal oxide nanoparticles were clearly different, several genes were identified to share a similar expression pattern among different treatment groups. The gene CJH_08660 that encodes a protein for integral cell membrane was upregulated in both ajoene-treated group and Al_2_O_3_ nanoparticles-treated group (**Supplementary Table S4**). In addition, the ajoene-treated group shared two upregulated genes with TiO_2_ nanoparticles-treated group, which were CJH_08660 (encoding an integral membrane protein) and CJH_02460 (encoding 50S ribosomal protein L24). We noticed that gene CJH_08660 was upregulated in all three individual treatment groups (i.e., ajoene, Al_2_O_3_ nanoparticles, and TiO_2_ nanoparticles), indicating all individual treatment could influence the integrity of cell membrane.

The differentially expressed genes (i.e., CJH_03855 and CJH_00775) induced by the combinatorial treatment of ajoene and Al_2_O_3_ nanoparticles were unique, as they were not detected in the ajoene-treated group or Al_2_O_3_ nanoparticles-treated group. Combinatorial treatment of ajoene and TiO_2_ nanoparticles induced only one differentially expressed gene (CJH_08660), which was detected in all individual treatment groups.

### Variations in Biochemical Compositions of *C. jejuni* Cells to Antimicrobial Treatment Using Confocal Micro-Raman Spectroscopy

**FIGURE 4:**
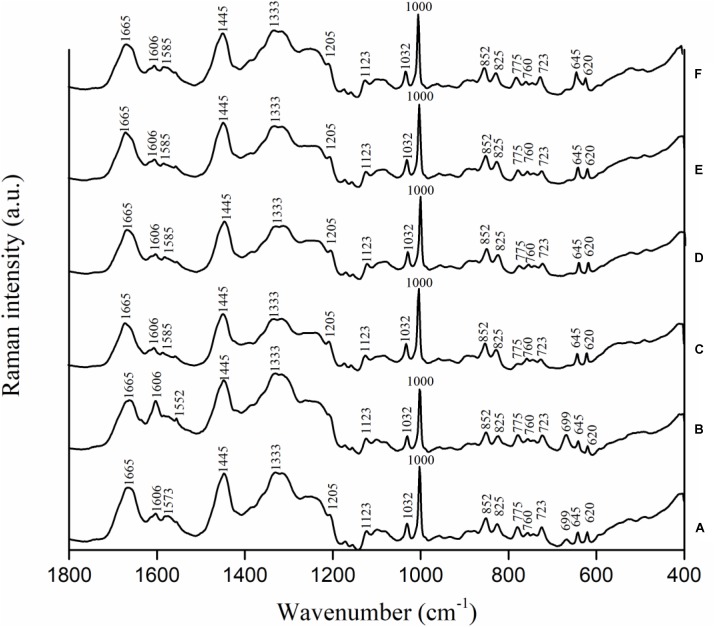
Biochemical compositional variations of *C. jejuni* F38011 cells caused by antimicrobial treatments were monitored using confocal micro-Raman spectroscopy. *C. jejuni* F38011 cells were treated with different antimicrobials (i.e., 1 mM ajoene, 16 mM TiO_2_ nanoparticles, 16 mM Al_2_O_3_ nanoparticles, 0.06 mM ajoene and 4 mM TiO_2_ nanoparticles, and 0.06 mM ajoene and 4 mM Al_2_O_3_ nanoparticles) for 1 h at 37°C prior to Raman spectral collection. **(A)** Average Raman spectrum of untreated sample. **(B)** Average Raman spectrum of 1 mM ajoene treated sample. **(C)** Average Raman spectrum of 16 mM Al_2_O_3_ nanoparticle treated sample. **(D)** Average Raman spectrum of 0.06 mM ajoene and 4 mM Al_2_O_3_ nanoparticle treated sample. **(E)** Average Raman spectrum of 16 mM TiO_2_ nanoparticle treated sample. **(F)** Average Raman spectrum of 0.06 mM ajoene and 4 mM TiO_2_ nanoparticle treated sample (*n* = 3).

*Campylobacter jejuni* strain F38011 was used as the representative strain to investigate the variations in biochemical compositions of bacterial cells caused by the antimicrobial treatments. *C. jejuni* cells were treated with different antimicrobials (i.e., 16 mM TiO_2_ nanoparticles, 16 mM Al_2_O_3_ nanoparticles, 0.06 mM ajoene and 4 mM TiO_2_ nanoparticles, and 0.06 mM ajoene and 4 mM Al_2_O_3_ nanoparticles) at 37°C with constant shaking for 1 h prior to Raman spectral collection. Untreated *C. jejuni* cells showed the prominent Raman peaks at 620, 645, 669, 723, 760, 775, 825, 852, 1,000, 1,032, 1,123, 1,205, 1,333, 1,445, 1,573, 1,606, and 1,665 cm^-1^ (**Figure 4A**). Raman peak assignments are summarized in **Supplementary Table S3**.

Compared to Raman spectral pattern of the untreated group, the average Raman spectrum derived from ajoene treated group did not show the peaks at 1,205 and 1,573 cm^-1^, but demonstrated a new prominent peak at 1,552 cm^-1^ (**Figure 4B**). In addition, the intensities of Raman peaks at 669, 1,333, and 1,445 cm^-1^ were higher of the ajoene treatment group than that of the untreated group. The peaks at 669, 1,333, and 1,573 cm^-1^ were derived from C-S stretching modes of cytosine, guanine, and guanine or adenine, respectively, which were all assigned as nucleic acids. The peaks at 1,205 and 1,552 cm^-1^ were related to proteins and *ν*(C = C) of tryptophan as the protein profiles. The peak at 1,445 cm^-1^ was associated with proteins and phospholipid from proteins and/or lipids. The absence of Raman peak at 1,205 and 1,573 cm^-1^ indicated that ajoene attached and disrupted the outer membrane protein of *C. jejuni* cells. The emergence of the prominent Raman peak at 1,552 cm^-1^ indicated the formation of disulfide bonds derived from the interaction between ajoene and bacterial outer membrane proteins. The increased intensity of Raman peaks at 669 and 1,333 cm^-1^ indicated that ajoene could penetrate cell membrane and bind with the intracellular nucleic acids. In general, ajoene could interact with membrane proteins and disrupt membrane structure.

The individual metal oxide nanoparticle treatment and combinatorial treatment did not cause remarkable variations in the biochemical compositions of *C. jejuni* cells. Raman spectra derived from individual metal oxide nanoparticle treatment and combinatorial treatment shared similar spectral patterns as that derived from the untreated sample. Compared to Raman spectral pattern derived from the untreated group, Raman spectra derived from individual metal oxide nanoparticle treatment and combinatorial treatment did not show the peaks at 669 and 1,573 cm^-1^, but demonstrated a new prominent peak at 1,585 cm^-1^ (**Figures 4C–F**). The peaks at 669 and 1,573 cm^-1^ were derived from C-S stretching modes of cytosine, guanine, and adenine assigned as nucleic acids. The Raman peak at 1,585 cm^-1^ was derived from C = C olefinic stretch assigned as proteins. The emergence of Raman peak at 1,585 cm^-1^ indicated the disruption of membrane proteins and the absence of Raman peaks at 669 and 1,573 cm^-1^ indicated bacterial cell lysis. In general, individual metal oxide nanoparticle treatment and combinatorial treatment did not cause remarkable variations in biochemical compositions of *C. jejuni* cells. We therefore concluded that individual metal oxide nanoparticle treatment and combinatorial treatment mainly physically disrupt bacterial cells.

To investigate the minor chemical variations caused by antimicrobial treatments, second-derivative transformation was applied on Raman spectra. The area and height of each Raman peak were calculated. However, there were no significant (*P* < 0.05) peak variations found (**Supplementary Figure S3**).

### Morphological Variations of *C. jejuni* Cells Caused by Antimicrobial Treatment

The influence of antimicrobial treatment on the morphology of *C. jejuni* cells was investigated using SEM. *C. jejuni* strain F38011 was used as the representative strain. *C. jejuni* cells were treated with different antimicrobials (i.e., 16 mM TiO_2_ nanoparticles, 16 mM Al_2_O_3_ nanoparticles, 0.06 mM ajoene and 4 mM TiO_2_ nanoparticles, and 0.06 mM ajoene and 4 mM Al_2_O_3_ nanoparticles) at 37°C for 1 h prior to SEM characterization. Untreated *C. jejuni* cells demonstrated the integral cellular structure with a characteristic helical shape (**Supplementary Figure S4A**). Both individual treatment and combinatorial treatment caused remarkable morphological change of *C. jejuni* cells, including cell deformation, loss of cell membrane integrity, and cell lysis (**Supplementary Figures S4B–E**). Morphological changes caused by the combinatorial treatments were identical to that by the individual treatments.

## Discussion

Developing new antimicrobial strategies are critical to reducing the emergence and prevalence of antibiotic resistance in the environment and food products. In the current study, we applied both organosulfur compounds (ajoene) and metal oxide nanoparticles (Al_2_O_3_ and TiO_2_ nanoparticles) to inactivate a leading foodborne pathogen *C. jejuni*. The individual treatment of ajoene or metal oxide nanoparticles all demonstrated the strong antimicrobial effect against *C. jejuni* when the appropriate concentration was applied. In addition, the antimicrobial efficacy was enhanced at a higher temperature (**Figures 2**, 3).

Researchers have found that the combinatorial treatment of several complementary antimicrobial compounds could generate a better antimicrobial effect. Li et al. (2005) identified that silver nanoparticles and amoxicillin could generate a synergistic antimicrobial effect against *E. coli*. They concluded that silver nanoparticles were the major antimicrobial components in the synergistic treatment (Li et al., 2005). In our previous study, ajoene was synthesized and showed a significant antimicrobial effect against *C. sakazakii* at a low concentration (3.88 mM). The mechanism of action of ajoene was proposed as the penetration of bacterial cell membrane and subsequent alteration of the conformational structure of thiol-containing proteins (Feng et al., 2014), which was complementary to the mechanism of action of metal oxide nanoparticles. Hence, the combination of ajoene and metal oxide nanoparticles theoretically could generate a synergistic antimicrobial effect. In the current study, we identified that a combinatorial treatment of ajoene and metal oxide nanoparticles could generate a synergistic antimicrobial effect against *C. jejuni*. The synergy was achieved when the concentration of metal oxide nanoparticles was higher than 0.5 mM. The synergistic treatment could maximize the antimicrobial effect of each individual compound. A higher concentration of metal oxide nanoparticles in the combinatorial treatment could enhance the antimicrobial efficacy.

To investigate the antimicrobial mechanism of the synergistic treatment, the transcriptional response of *C. jejuni* to each individual treatment was determined using RNA-seq analysis. The treatment of ajoene induced a range of transcriptional responses in *C. jejuni*. The upregulated genes are mainly involved in two pathways responsible for transcription-translation and ATP utilization (**Table 1**). Besides, several stress response genes, including *dnak*, *groEL*, *katA*, *cmeA*, *cmeB*, and *cmeC*, were also upregulated (**Supplementary Table S4**). *Dnak* and *groEL* are general stress response genes that mediate heat and starvation tolerance (Konkel et al., 1998; Klančnik et al., 2006) and *cmeA*, *cmeB*, and *cmeC* together encode an efflux pump that mediates the intrinsic tolerance to a broad range of antimicrobials (Lin et al., 2002; Gibreel et al., 2007). The *katA* gene mediates the tolerance of bacterial cells to oxidative stress (Grant and Park, 1995; Van Vliet et al., 1999). On the other hand, downregulated genes are mainly involved in a pathway responsible for the integrity of cell membranes. In addition, a DNA repair-associated gene *recN* and a chemotaxis-associated gene *cheY* were also significantly downregulated (**Supplementary Table S4**). All these transcriptional profiles indicated that ajoene treatment at sub-lethal concentration induced mild stress rather than immediate inactivation of *C. jejuni*. The antimicrobial mechanism of ajoene has been proposed as the inhibition of thiol-containing enzymes via the interaction with thiol groups (Ankri and Mirelman, 1999; Ilić et al., 2011) and this interaction is reversible due to its non-covalence feature. Thiol groups, which have been also extensively identified from the membrane proteins or cell wall-bound proteins, serve as the reducing compounds to protect bacterial cell from oxidative stress (Navarre and Schneewind, 1999; Möller and Hederstedt, 2006; Michelon et al., 2010). Therefore, the interaction between ajoene and thiol groups of membrane proteins can decrease the integrity of cell membrane and elevate the oxidative state. In agreement with these studies, our RNA-seq results and Raman spectroscopic analyses demonstrated that ajoene treatment for a short period (1 h) at a sub-lethal concentration (1 mM) could disrupt membrane proteins by forming disulfide bonds and trigger a series of stress responses in *C. jejuni* (**Figures 1, 4**). The treatment of ajoene could stimulate metabolic activities of transcription-translation and ATP utilization indicated as upregulation of RNA binding and ATP binding associated genes, but inhibit the synthesis of proteins responsible for integral cell membrane and chemotaxis indicated as downregulation of the cell membrane- and chemotaxis-associated genes (**Table 1**). The treatment of ajoene could penetrate bacterial cell membrane and interact with either the outer membrane proteins or intracellular nucleic acids (**Figure 4** and **Supplementary Table S3**). All these transcriptional and biochemical profiles of bacterial cells indicated that ajoene treatment might increase the susceptibility of *C. jejuni* to stress by impairing the integrity of cell membrane and chemotaxis of the cells.

In this study, treatment of metal oxide nanoparticles (Al_2_O_3_ nanoparticles and TiO_2_ nanoparticles) alone rarely induced any detectable transcriptional response of *C. jejuni*. The treatment of Al_2_O_3_ nanoparticles only induced two differentially expressed genes and the treatment of TiO_2_ nanoparticles induced seven differentially expressed genes, including four upregulated genes with the similar function to maintain the integrity of cell membrane. Previous studies proposed several antimicrobial mechanisms for metal oxide nanoparticles. The adhesion and subsequent disruption of bacterial cell membrane were believed to be one of the major antimicrobial mechanisms of metal oxide nanoparticles. According to Li and Logan (2004), Al_2_O_3_ nanoparticles were determined to have a positive surface charge at neutral pH that could interact with the negatively charged surface of *E. coli* cells through electrostatic interaction. In another study, Pakrashi et al. (2011) confirmed this statement and found that Al_2_O_3_ nanoparticles coagulated at the cell membrane, disrupted cellular structure and subsequently resulted in the rupture of *Bacillus licheniformis* cells. TiO_2_ nanoparticles were proposed to have a photo-killing mechanism. It has been found that UV illumination could enhance the antimicrobial efficacy of TiO_2_ nanoparticles against a variety of bacteria, including *P. aeruginosa*, *E. coli*, and *S. aureus* (Maness et al., 1999). Photocatalysis of TiO_2_ nanoparticles could induce the peroxidation of bacterial cell membrane, resulting in the disruption of cell respiration (Foster et al., 2011). Our current study illustrated that TiO_2_ nanoparticles treatment could generate a significant antimicrobial effect against *C. jejuni* even without UV illumination (**Figures 2A, 3A**). Based on this observation, we speculated that the antimicrobial mechanism of TiO_2_ nanoparticles might be on the basis of adhesion and physical disruption of bacterial cell membrane, which was a universal antimicrobial mechanism shared by different types of metal oxide nanoparticles. The induction of ROS was also proposed as one of the antimicrobial mechanisms of metal oxide nanoparticles. The previous study found the treatment of ZnO nanoparticles could induce the oxidative stress response in *C. jejuni* and resulted in a significant upregulation of stress response genes (Xie et al., 2011). However, we did not observe any gene associated with oxidative stress in response to either Al_2_O_3_ nanoparticle or TiO_2_ nanoparticle treatment in this study.

The combinatorial treatment of ajoene and metal oxide nanoparticles barely induced any transcriptional response in *C. jejuni*. The combinatorial treatment of Al_2_O_3_ nanoparticles and ajoene only induced two differentially expressed genes (CJH_03855 and CJH_00775) and these two genes were not shown in any of the individual treatment groups. The combinatorial treatment of TiO_2_ nanoparticles and ajoene only induced one differentially expressed gene (CJH_08660) that were presented in all individual treatment groups. Hence, the transcriptional response of *C. jejuni* to combinatorial treatment shared more similarity to that of individual metal oxide nanoparticle treatment. Raman spectroscopic analysis was consistent to the results of transcriptional analysis. Raman spectra of combinatorial treated samples shared similar patterns to that of metal oxide nanoparticle treated samples (**Figure 4**). For example, the peak at 699 cm^-1^ was prominent in Raman spectra of untreated and ajoene treated samples, but absent from Raman spectra of metal oxide nanoparticle treated and combinatorial treated samples. The peak at 1,606 cm^-1^ demonstrated a high intensity in Raman spectra of ajoene treated sample, but a relatively low intensity in Raman spectra of metal oxide nanoparticle treated and combinatorial treated samples. We concluded that metal oxide nanoparticles were the leading antimicrobial factor in the combinatorial treatment. According to the specific antimicrobial mechanisms of ajoene and metal oxide nanoparticles, we proposed a two-phase antimicrobial mechanism for synergistic treatment, namely, inducing phase mediated by ajoene and reaction phase mediated by metal oxide nanoparticles. The treatment of ajoene caused damage to cell membrane and subsequently increased the susceptibility of *C. jejuni* to the stress. In the meanwhile, the treatment of metal oxide nanoparticles adhered to the injured cell membrane and physically disrupt *C. jejuni* cells, as observed by SEM (**Supplementary Figure S4**).

In this study, we developed a novel and practical intervention strategy to inactivate *C. jejuni*. Ajoene and metal oxide nanoparticles were identified as the major antimicrobials to generate a synergistic effect and they are considered as generally safe. Immobilization and conjugation of these nanoparticles and ajoene on food packaging materials and food processing contact surfaces can be a potential application of this synergistic antimicrobial treatment used in food industry.

## Author Contributions

XL, SW, and MK were responsible for experimental design and manuscript editing. RX, JF, LM, CL, and MX performed the experiments. RX and JF analyzed the data and drafted the manuscript.

## Conflict of Interest Statement

The authors declare that the research was conducted in the absence of any commercial or financial relationships that could be construed as a potential conflict of interest.
