# Supplementary material for: Whole Transcriptome Sequencing Analysis of the Synergistic Antimicrobial Effect of Metal Oxide Nanoparticles and Ajoene on Campylobacter jejuni
Source: Front Microbiol. 2018 Aug 31;9:2074. doi: 10.3389/fmicb.2018.02074 (PMC6127312; doi:10.3389/fmicb.2018.02074)
Supplement: Supplementary file 1 [file Table_1.docx]

**Supplementary material**

**

**

**Fig S1.** The gene expression profile of *C. jejuni* F38011 from RNA-seq analysis was validated by qPCR. A total of 7 differentially expressed genes derived from different treatment groups (1 mM ajoene, 16 mM Al_2_O_3_ nanoparticles, 16 mM TiO_2_ nanoparticles, 0.06 mM ajoene and 4 mM Al_2_O_3_ nanoparticles, and 0.06 mM ajoene and 4 mM TiO_2_ nanoparticles) were selected as the representatives.


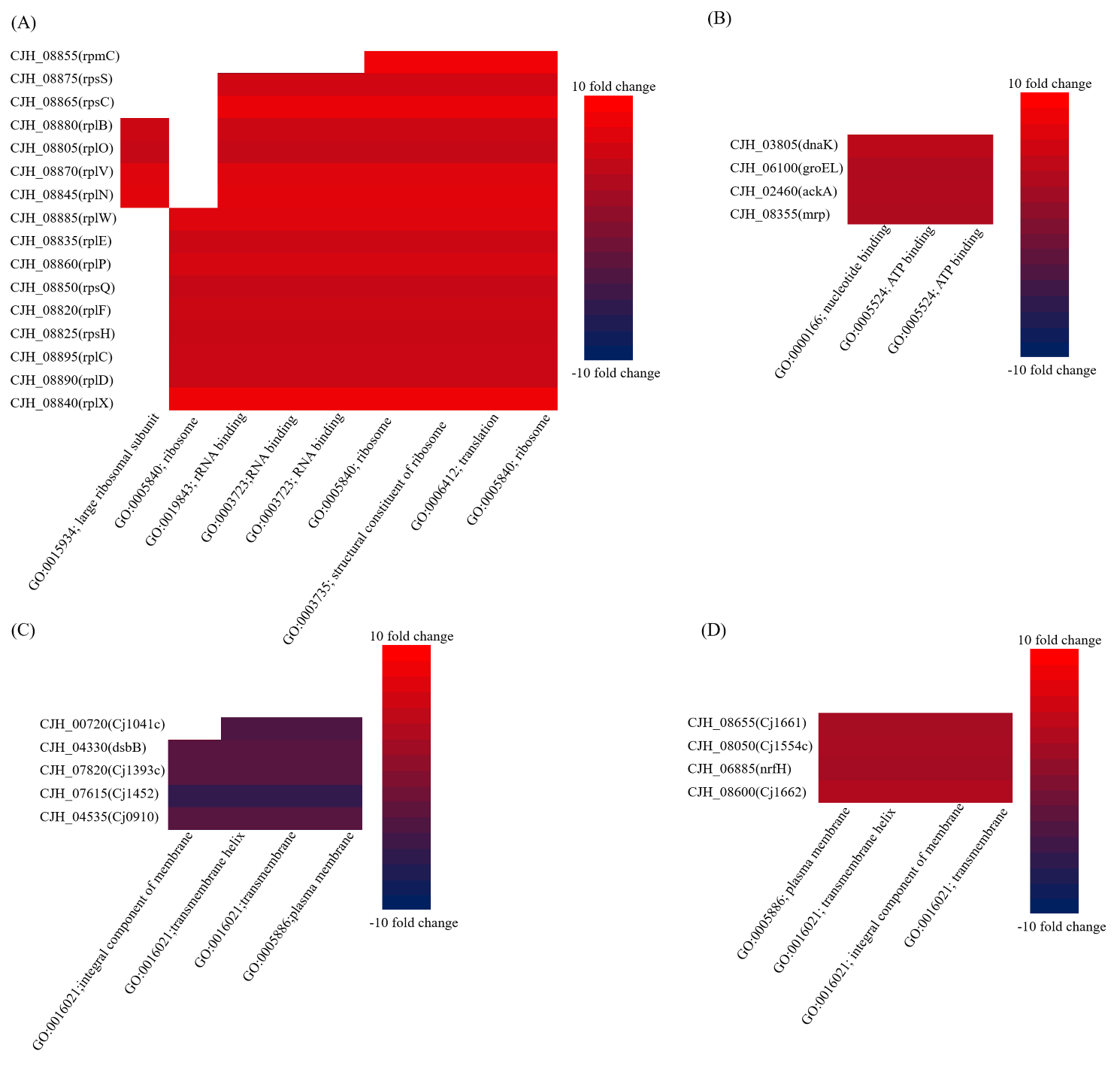


**Fig S2.** Transcriptional response of *C. jejuni* F38011 cells to the treatment of ajoene and metal oxide nanoparticles is categorized based on the functional terms. Panels: (A) Up-regulated genes induced by the treatment of 1 mM ajoene were clustered on the basis of transcription-translation term; (B) Up-regulated genes induced by the treatment of 1 mM ajoene were clustered on the basis of energy utilization term; (C) Down-regulated genes induced by the treatment of 1 mM ajoene were clustered on the basis of integral cell membrane term; and (D) Up-regulated genes induced by the treatment of 16 mM TiO_2_ were clustered on the basis of integral cell membrane term. The clusters shown in white color in panels (A) and (C) indicate that the differentially expressed genes were absent in certain functional terms (n = 3).


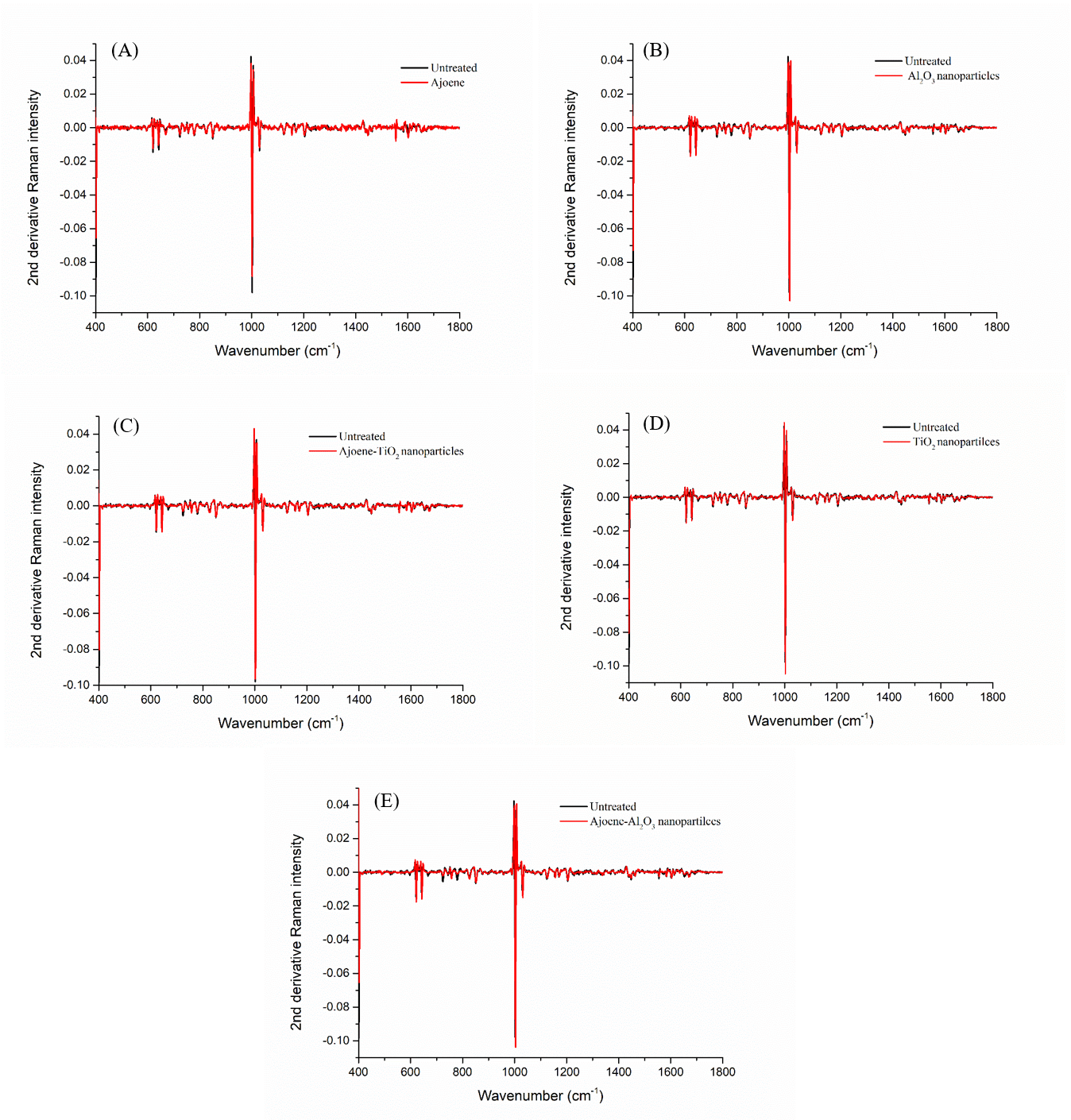


**Fig S3.** Second-derivative transformation of Raman spectra for the comparison of (A) ajoene treated sample with untreated sample; (B) Al_2_O_3_ nanoparticles treated sample with untreated sample; (C) Al_2_O_3_ nanoparticles-ajoene treated sample with untreated sample; (D) TiO_2_ nanoparticles treated sample with untreated sample; (E) TiO_2_ nanoparticles-ajoene treated sample with untreated sample (n = 3).


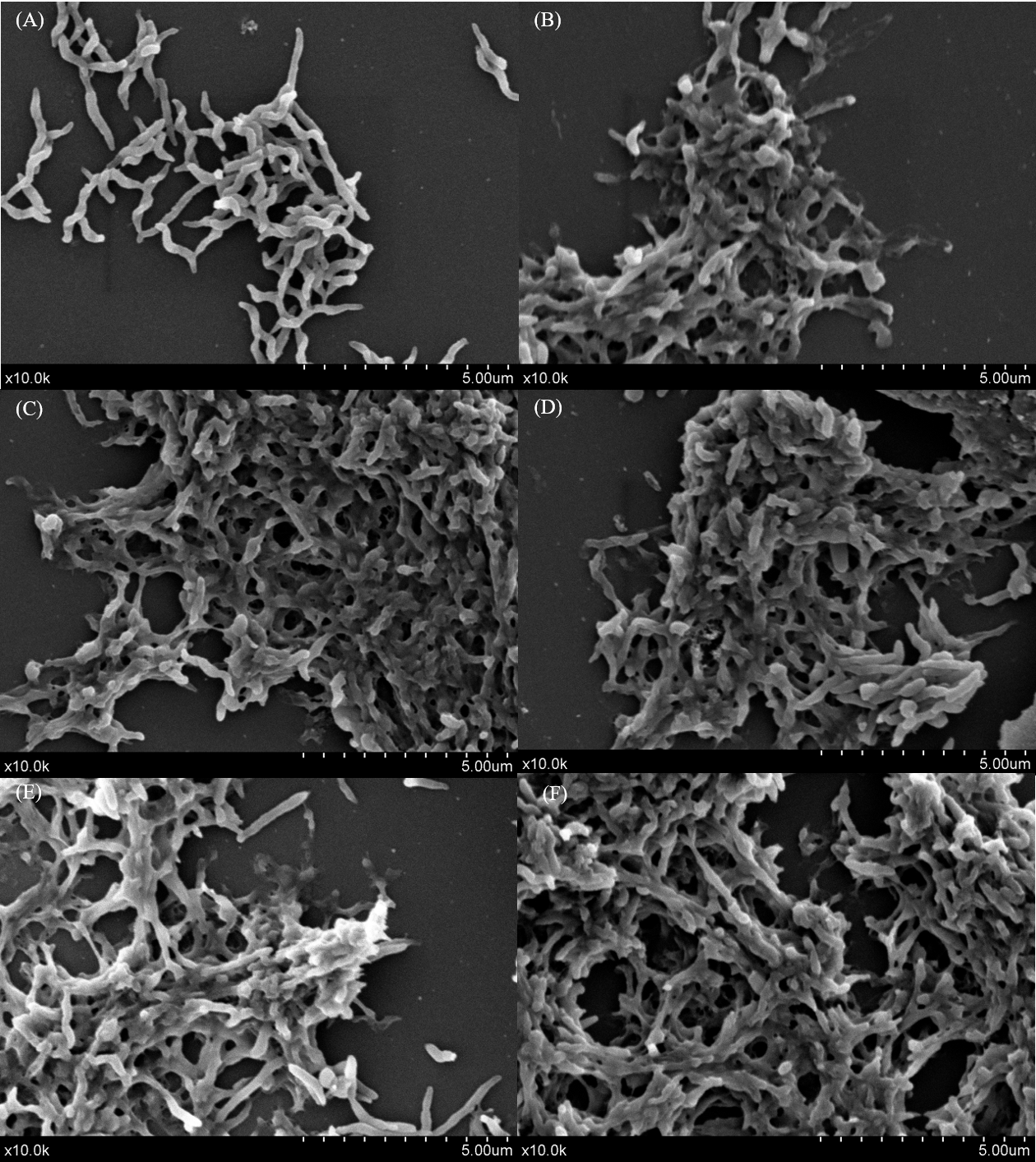


**Fig S4.** Scanning electron microscopic analysis of the morphological variations of *C. jejuni* F38011 cells with different antimicrobial treatments. (A) Untreated cells. (B) Ajoene treated cells. (C) Al_2_O_3_ treated cells. (D) Al_2_O_3_-ajoene treated cells. (E) TiO_2_ treated cells. (F) TiO_2_-ajoene treated cells.

**Table S1.** Bacterial strains and plasmids used in the current study.

| Strains | Description | | Reference |
| --- | --- | --- | --- |
| *C. jejuni* F38011 | human clinical isolate | ([Feng et al., 2016](#_ENREF_1)) | |
| *C. jejuni* ATCC 33560 | product quality control strain | ATCC | |
| *C. jejuni* y110539 | human clinical isolate | Laboratory collection obtained from Dr. Michael Konkel (Washington State University) | |
| *C. jejuni* z110526 | human clinical isolate | Laboratory collection obtained from Dr. Michael Konkel (Washington State University) | |

**Table S2.** The primers used for qPCR validation

| Primer | Sequence (5’-3’) |
| --- | --- |
| *rpoA* RT-F | CGAGCTTGCTTTGATGAGTG |
| *rpoA* RT-R | AGTTCCCACAGGAAAACCTA |
| *slyD* RT-F | TGCGGTTCAAACTTTACCAA |
| *slyD* RT-R | GTTTCGCCATTTTCACCTTC |
| CJH_03855 (Cj0768) RT-F | GGGTACGTGAAATGCCTTTT |
| CJH_03855 (Cj0768) RT-R | AGCTTGCGATAATAGGAGGG |
| CJH_05030 (Cj1004) RT-F | ACAACATAATTTCAAAGCGCC |
| CJH_05030 (Cj1004) RT-R | TTTCCTTCCAAGCTCCATCT |
| CJH_02460 (Cj0689) RT-F | ATGCTTGAAAGTGCTGCAAA |
| CJH_02460 (Cj0689) RT-R | GGATTAGCACTAAGTCCGCT |
| CJH_08660 (Cj1662) RT-F | CGTGGTGCAAAATTTAAGCG |
| CJH_08660 (Cj1662) RT-R | TGATTGCTTGTGTTTTGGCT |
| CJH_07030 (Cj1385) RT-F | AGTCTTGTGCCTTTGATGGA |
| CJH_07030 (Cj1385) RT-R  CJH_02840 (Cj0561) RT-F  CJH_02840 (Cj0561) RT-R  CJH_08840 (Cj0439) RT-F  CJH_08840 (Cj0439) RT-R | GGACTAAAGGCAGCTTGTTC  TTCCTGTGTTTTAGCCTCCA  TAATATCCCTTGCACCCACA  AGCGGAAGAGATGAAAATGC  GCGTTCTTTTGCACCCTTAT |

**Table S3.** Raman band assignments for *C. jejuni* cells ([Naumann, 2001](#_ENREF_3);[Movasaghi et al., 2007](#_ENREF_2);[Talari et al., 2015](#_ENREF_4)).

| Raman shift (cm^-1^) | Band assignment |
| --- | --- |
| 620 | C-C twist aromatic ring |
| 645 | C-C twisting mode of phenylalanine (proteins) |
| 669 | C-S stretching mode of cytosine |
| 723 | Nucleic acids band |
| 760 | Ring breathing tryptophan (proteins) |
| 775 | Phosphatidylinositol (lipids) |
| 825 | Phosphodiester (DNA) |
| 852 | Proline, hydroxyproline, tyrosine |
| 1000 | Phenylalanine |
| 1032 | CH_2_CH_3_ bending modes of collagen and phospholipids |
| 1123 | (C-N), proteins; C-C stretching mode of lipids and proteins |
| 1205 | protein content |
| 1333 | Guanine |
| 1445 | *δ*(CH_2_), *δ*(CH_3_) (protein assignment); CH_2_CH_3_ bending modes of proteins and phospholipids |
| 1552 | *ν*(C=C), tryptophan (protein assignment) |
| 1573 | Guanine, adenine |
| 1585 | C=C olefinic stretch (protein assignment) |
| 1606 | C=C bending protein assignment |
| 1665 | Amide I |

**References**

Feng, J., Lamour, G., Xue, R., Mirvakliki, M.N., Hatzikiriakos, S.G., Xu, J., Li, H., Wang, S., and Lu, X. (2016). Chemical, physical and morphological properties of bacterial biofilms affect survival of encased *Campylobacter jejuni* F38011 under aerobic stress. *Int*.*J*. *Food. Microbiol.* 238**,** 172-182. [doi.org/10.1016/j.ijfoodmicro.2016.09.008](https://doi.org/10.1016/j.ijfoodmicro.2016.09.008)

Movasaghi, Z., Rehman, S., and Rehman, I.U. (2007). Raman spectroscopy of biological tissues. *‎Appl. Spectrosc. Rev.* 42, 493-541. [doi.org/10.1080/05704920701551530](https://doi.org/10.1080/05704920701551530)

Naumann, D. (2001). FT-infrared and FT-Raman spectroscopy in biomedical research. ‎*Appl. Spectrosc. Rev.* 36, 239-298. [doi.org/10.1081/ASR-100106157](https://doi.org/10.1081/ASR-100106157)

Talari, A.C.S., Movasaghi, Z., Rehman, S., and Rehman, I.U. (2015). Raman spectroscopy of biological tissues. ‎*Appl. Spectrosc. Rev.* 50, 46-111. [doi.org/10.1080/05704928.2014.923902](https://doi.org/10.1080/05704928.2014.923902)
